# Characterization of Flagellotropic, Chi-Like *Salmonella* Phages Isolated from Thai Poultry Farms

**DOI:** 10.3390/v11060520

**Published:** 2019-06-05

**Authors:** Preeda Phothaworn, Matthew Dunne, Rattaya Supokaivanich, Catherine Ong, Jiali Lim, Rutjawate Taharnklaew, Mongkol Vesaratchavest, Rabuesak Khumthong, Onanong Pringsulaka, Pravech Ajawatanawong, Jochen Klumpp, Nathan Brown, Mohammed Imam, Martha R. J. Clokie, Edouard E. Galyov, Sunee Korbsrisate

**Affiliations:** 1 Department of Immunology, Faculty of Medicine Siriraj Hospital, Mahidol University, Bangkok 10700, Thailand; ping_medtech@hotmail.com (P.P.); setepenre004@hotmail.com (R.S.); 2 Institute of Food, Nutrition and Health, ETH Zurich, Zurich 8092, Switzerland; mdunne@ethz.ch (M.D.); jochen.klumpp@hest.ethz.ch (J.K.); 3 DSO National Laboratories, Singapore 117510, Singapore; catong@dso.org.sg (C.O.); ljiali@dso.org.sg (J.L.); 4 Betagro Science Center Co., Ltd., Pathumthani 12120, Thailand; rutjawate@betagro.com (R.T.); mongkolv@betagro.com (M.V.); rabuesakk@betagro.com (R.K.); 5 Department of Microbiology, Faculty of Science, Srinakharinwirot University, Bangkok 10110, Thailand; onanong@swu.ac.th; 6 Department of Microbiology, Faculty of Science, Mahidol University, Bangkok 10400, Thailand; pravech.aja@mahidol.ac.th; 7 Department of Genetics and Genome Biology, University of Leicester, Leicester LE1 7RH, UK; nmb22@leicester.ac.uk (N.B.); mi135@le.ac.uk (M.I.); mrjc1@le.ac.uk (M.R.J.C.)

**Keywords:** bacteriophage, Chi-like virus, flagellotropic, *Salmonella*, siphovirus, poultry farm, prevalent, phylogenomic tree

## Abstract

Despite a wealth of knowledge on *Salmonella* phages worldwide, little is known about poultry-associated *Salmonella* phages from Thailand. Here, we isolated 108 phages from Thai poultry farms that infect *Salmonella enterica* serovar Typhimurium. Phages STm101 and STm118 were identified as temperate *Siphoviridae* phages. Genome sequencing and analyses revealed these phages share approximately 96% nucleotide sequence similarity to phage SPN19, a member of the Chi-like virus genus. PCR amplification of the gene encoding capsid protein E of the Chi-like phage was positive for 50% of phage isolates, suggesting a predominance of this phage type among the sampled poultry farms. In addition to the flagella, two phages required the lipopolysaccharide to infect and lyse *Salmonella.* Furthermore, phylogenomic analysis demonstrated that phages STm101 and STm118 formed a monophyletic clade with phages isolated from Western countries, but not from closer isolated phages from Korea. However, further investigation and more phage isolates are required to investigate possible causes for this geographic distribution.

## 1. Introduction

Bacteriophages (phages) are viruses that infect bacteria, and play important roles in regulating abundance, diversity, and composition of bacterial communities [1]. A significant number of *Salmonella* phages have been isolated and characterized from diverse environments. This includes sequencing of complete genomes from a wide variety of dsDNA, tailed phages of the order *Caudovirales* [2,3]. *Salmonella* phages are exploited in various lab-based applications including strain construction via transduction [4,5,6] and phage typing for epidemiological purposes [7,8]. The inherent *Salmonella*-specificity of certain phages (and their component proteins) has also been exploited to develop highly specific bio-probes for the detection of foodborne *Salmonella* [9,10,11,12] and as antibiotic alternatives for eliminating *Salmonella* in food production [13,14,15,16].

The *Siphoviridae Salmonella* phage χ (Chi) was first isolated by Sertic and Boulgakov in 1936 [17]. It is a flagellotropic phage and attaches to the flagella of motile bacteria as its primary receptor [17]. Due to the relatively low likelihood of encounters between phages and bacteria in certain environments, it has been proposed that phage Chi, as well as others [18,19,20], recognize motile flagella and/or pili to increase the probability of interacting with a metabolically active host suitable for infection [19]. The genomic sequence of phage Chi is approximately 59 kb long with 56.5% GC content and 75 open reading frames (ORFs) [21,22]. Related Chi-like phages with similar genome sizes, gene contents, and gene orders to phage Chi include *Salmonella* phages FSLSP030, FSL_SP-039, FSLSP088, FSL_SP-124, and SPN19 [21,23], *Enterobacter cancerogenus* phage Enc34 [24], and *Providencia stuartii* phage RedJac [25]. Additionally, non-Chi-like flagellotropic phages have been identified such as *Caulobacter crescentus* phages ϕCb13 and ϕCbK [19], the Gram-positive targeting *Bacillus subtilis* phage PBS1 and *Bacillus pumilus* phage PBP1 [26], and more recently the *Agrobacterium* phage 7-7-1 [27]. The broad host ranges typically observed for flagellotropic phages (e.g., phage Chi spans *Salmonella* and *Escherichia coli* hosts [28,29]) makes them attractive candidates for development into tools for pathogen detection and remediation.

Here, we describe the isolation of 108 *Salmonella* phages from chicken fecal and boot swap samples of Thai poultry farms. Over 50% of the phages were identified as members of the Chi-like genus, suggesting a predominance of this phage type within Thai poultry farms. Two phages from the same farm were randomly chosen for further characterization using transmission electron microscopy (TEM) and whole genome sequencing. The phylogenomic relationship among these newly isolated Thai Chi-like phages and other available reported Chi-like phages was also investigated. Finally, using *S.* Typhimurium (*S*. Tm) outer membrane mutants, we identified that in addition to the flagella, these Chi-like phages required the lipopolysaccharide (LPS) for efficient host attachment and infection.

## 2. Materials and Methods

### 2.1. Bacterial Strains and Media

All strains used for host range analysis and mutant studies are listed in Table 1, Table 3, and Supplementary Table S1. *S*. Tm MA8508 was used for *Salmonella* phage isolation and propagation from fecal and boot swab samples. Cells were grown at 37 °C in trypticase soy broth (TSB) or agar (TSA) (Titan Biotech, Delhi, India). Saline magnesium (SM) buffer (50 mM Tris-HCl, pH 7.5, 100 mM NaCl, 8.0 mM MgSO_4_, and 0.01% gelatin) was used for phage storage at 4 °C.

### 2.2. Isolation, Purification, and High Titer Phage Stock Preparation

With the owners’ authorization, 252 fecal samples were obtained from four free-range chicken farms dispersed across the Lamphun province, Northern Thailand, and 169 boot swab samples were collected from staff working at a commercial farm in central Thailand. Each sample was screened for the presence of phages using spot assays as described [30]. Briefly, 2 g of fecal sample was suspended in SM buffer then centrifuged at 4500× *g* for 30 min while boot swab sample in buffered peptone water (Merck, Branchburg, New Jersey, USA) was centrifuged at 4500× *g* for 10 min. Then the centrifuged supernatant was filtered through a 0.45 µm membrane filter (GE Healthcare Life Sciences, Marlborough, MA, USA). Ten microliters of the filtrated suspension were spotted onto a bacterial lawn containing 100 μL of a mid-log phase *S.* Tm MA8508 culture mixed with 3 mL soft agar, and incubated overnight at 37 °C. Samples producing plaques (zones of localized lysis) were confirmed to contain phages using the double agar overlay plaque technique as previously described [31]. Single plaques were then picked and purified a minimum of five times by repeated double agar overlays. 

For high titer phage stock production, double agar overlays were used to produce ten semi- confluent lysis plates (diameter 150 mm) from a single plaque of an isolated phage. Plates were gently agitated at room temperature with SM buffer for 5 hours to produce a crude phage lysate. The lysate was centrifuged at 6000× *g* for 45 min at 4 °C, and the supernatant filtered through a 0.45 µm membrane filter, before a final centrifugation at 18,000× *g* for 30 min at 4 °C. The supernatant was gently removed, and 2 mL of SM buffer added to the pellet and incubated overnight at 4 °C. On the next day, the phage suspension was centrifuged at 1800× *g*, for 15 min at 4 °C. For each phage, a high titer of approximately 10^11^ pfu/mL (plaque forming units/mL) was recovered and stored at 4 °C.

### 2.3. Host Range Analysis of Isolated Phages

Spot assays were also used to determine phage host range. Briefly, 10 μL of phage solution taken from a serial dilution was spotted onto a bacterial lawn containing 100 μL of mid-log phase bacterial culture mixed with 3 mL of 0.35% TSA supplemented with 5 mM CaCl_2_ (soft agar), and incubated overnight at 37 °C. The presence of a lytic zone was considered evidence of phage susceptibility or lysis from without due to a high multiplicity of infection (MOI) from direct spotting of undiluted phage solutions; no lysis was considered evidence of phage resistance. All negative results were confirmed again using double agar overlay plaque assay [30].

### 2.4. Amplification of Salmonella Chi-Like Phage Capsid Protein E DNA

The gene encoding the major capsid protein E of the Chi-like *Salmonella* phage SPN19 (GenBank accession number NC_019417.1) was used as a template to design two PCR primers: *forward* (5’-TTCAGACCCACGGATGGTTG-3’) and *reverse* (5’-AGAAAGCGGCTACAACACGA-3’), corresponding to nucleotide positions 41,881–41,830 and 42,302–42,321, respectively, on the complete genome. The PCR cycle composed of a hot start at 94 °C for 3 min, followed by 35 cycles of 94 °C for 1 min, 60 °C for 30 s, and 72 °C for 30 s, before a final extension of 72 °C for 5 min. The amplified DNA fragment was 511 bp in length and was detected after 1% agarose gel (Vivantis Technologies, Selangor Darul Ehsan, Malaysia) electrophoresis and stained with GelRed® nucleic acid gel stain (Biotium, Fremont, CA, USA).

### 2.5. One-Step Phage Growth Curve Analysis

One-step growth curves were performed as described [30]. Briefly, a culture of *S*. Tm MA8508 was grown to OD_600_ of 0.2–0.5 and mixed with individual phage suspensions at a multiplicity of infection (MOI) of 0.01. Phage adsorption was performed for 15 min at 37 °C, after which the suspension was filtered through a 0.45 μm membrane filter. The membrane filter was placed in 50 mL of TSB supplemented with 5 mM CaCl_2_ (Merck, New Jersey, USA) and incubated at 37 °C. Every 5 min, 2 × 500 µL of suspension was collected and aliquoted into separate tubes. Using double agar overlay plaque assay, the first aliquot was used for pfu/mL determination of “free phages” (after removal by centrifugation of bacterial cells) and the second aliquot was chloroform treated to release intracellular phages for the “total phage” count. Briefly, 10 μL of each aliquot was serially diluted, mixed with 100 μL of mid-log phase *S.* Tm MA8508, 3 mL of 0.35% TSA, and poured onto a TSA supplemented with 5 mM CaCl_2_. After overnight incubation at 37 °C, total pfu/mL was determined. Combining these numbers, the latent period, eclipse period, and burst size were calculated as a one-step growth curve.

### 2.6. Transmission Electron Microscopy

Transmission electron microscopy was performed as previously described [30]. Briefly, a high titer (>10^9^ pfu/mL) phage solution was dropped onto a 100-mesh copper grid coated with Formvar (Ted Pella, Redding, CA, USA) then negative stained with 2% potassium phosphotungstate (Sigma-Aldrich, St. Louis, MO, USA), pH 7.2. The grid was examined using a TEM-1230 transmission electron microscope (JEOL, Tokyo, Japan) equipped with a Dual vision digital camera (Gatan, Pleasanton, CA, USA). Phage morphology was classified based on order and family according to Ackermann [32,33].

### 2.7. Next-Generation Sequencing of Phage Genome and Sequences Analysis

Phage DNA was extracted by a phenol/chloroform extraction method as described [34]. Genome sequencing of phages were performed using the Ion Torrent PGM with Ion 314 V2 chips, following the manufacturer’s protocol for 200 bp genomic DNA fragment library preparation (Ion Xpress Plus gDNA fragment library system), template preparation (Ion OneTouch system), and sequencing (Ion PGM 200 sequencing kit) (Thermo Fisher Scientific, Waltham, MA, USA). De novo assembly of the reads was performed via succinct de Bruijn graph using MEGAHIT software version 1.2.x [35,36]. CAP3 Assembly Program was used to assemble the final phage contig and to remove the overlapping sequences [31]. To annotate the contigs, the genome sequences in FASTA format were annotated using Prokka version 1.2 [37]. The outputs of each phage were compared and manually curated against the reference *Salmonella* phage SPN19 (NC_019417.1) to obtain the final set of genes in each phage genome. Easyfig software version 2.2.3 was used to generate the phage alignment figure [38]. The completed genome sequences of *Salmonella* phages STm101 and STm118 are available from GenBank under accession numbers KX765862 and KX765863, respectively.

### 2.8. Comparison, Clustering, and Phylogenomic Analysis of Phage Genomes

Comparative genomics was performed using CLC Genomics Workbench (Version 12.0, QIAGEN) using *Salmonella* phage SPN19 (NC_019417.1) as a reference strain. Phage genome sequences were mapped to the reference using default parameters and the mapped reads were further analyzed for genome variation with the CLC Genomics Workbench. Only reads that have minimum coverage of 30X and frequency of more than 70% were included for the analyses. The 139 *Salmonella* phage genomes were downloaded from the GenBank database [39] and screened for completeness before being added to the analysis, as shown in Supplementary File 1. In addition, geographic areas of the selected *Salmonella* phages were summarized in Supplementary Table S2. Coding sequences were identified and translated from all genomes. Protein sequences from each genome were stored separately. Those protein sequences were compared all-versus-all with standalone blastp via a Perl script (“get_homologues.pl”) with the following command and options: get_homologues.pl -A -t 0 -e -z -G [40]. In brief, the Perl script used a “cluster of orthologous group” (COG) algorithm to identify and cluster sets of homologous protein sequences using an E-value threshold of 10^−5^. The in-paralogs found in the process were omitted. An all-versus-all matrix containing the average amino acid identity (AAI) of all protein coding sequences within each genome was generated. Pairwise distances from a correlation matrix built from all pairwise AAI values were hierarchically clustered using R-script [41] to identify phage genomes with significant similarity to *Salmonella* phage genomes. The command used was: hclust (as.dist (1 – cor (t (aai.matrix), use = “pairwise.complete.obs”)), method = “average”). 

Using COG analysis, a cluster of 14 related phages, including STm101 and STm118 were identified as sharing a core set of 28 orthologs. Protein multiple sequence alignments (MSAs) from these orthologs were built with default settings in MUSCLE v3.8.31 [42] and used to build codon-aware multiple alignments of the nucleotide sequences corresponding to each protein coding sequence with another Perl script (“pal2nal.pl”) [43]. The codon-aligned nucleotide sequences for the 28 orthologs in each phage genome were concatenated and used to build an unrooted phylogenomic tree in FastTree Version 2.1.10 [44] using the generalized time reversible nucleotide substitution model. The reliability of splits in the tree is estimated by local support values that are tested on 1000 resamples using the Shimodaira–Hasegawa method [45] on nearest-neighbor interchanges at each split.

## 3. Results

### 3.1. Host Range Analysis of the Two Thai Phages

In total, 252 chicken droppings from four farms and 169 boot swab samples from one farm were tested for phages that infect *S.* Tm MA8508. Overall, 108 samples produced lysis and formed single plaques, indicating phage activity. A single plaque (representing an individual phage) was picked from each of the 108 plaque-positive samples, passaged a minimum of five times, and then plaque morphology assessed. Three different phage morphologies were observed: (i) clear (29.6%); (ii) turbid (16.7%); and (iii) halo-forming plaques (53.7%), with halo-forming plaques accounting for over half of phages isolated. Seventeen phage isolates were randomly selected from the five farms for host range analysis using 44 different *Salmonella* strains, as shown in Supplementary Table S3. All phages presented different host ranges, including phages isolated from the same farm. The two clear plaque-forming phages isolated from the same farm named STm101 and STm118 were selected for further investigation.

The host ranges of the two phages were tested against a broader 118 strains of *Salmonella,* including 98 strains from the five serovars *S*. Enteritidis, *S*. Typhimurium, *S*. Virchow, *S.* Choleraesuis, and *S*. Hadar, as they are commonly associated with *Salmonella* infections of poultry plus 20 strains of other *Salmonella* serovars, as shown in Table 1. Overall, phages STm101 and STm118 were capable of infecting 39.8% (47/118) and 46.6% (55/118) of the tested strains, respectively. Phages STm101 and STm118 could lyse 9.4% (3/32) and 65.6% (21/32) of tested *S*. Enteritidis strains, respectively. None of the total 15 tested strains of *S*. Albany, *S*. I 4,[5],12:i:-, *S*. Altona, *S*. Corvallis, *S*. Give, *S*. Schwarzengrund, and *S*. Singapore could be infected by phages STm101 and STm118. Phage STm118, but not STm101, could infect a single *S.* Kentucky strain. None of the phages infected any of the other Gram-negative strains tested, which involved five *E. coli* strains and eight *Vibrio* spp. strains, or Gram-positive strains of *Staphylococcus aureus* and *Listeria monocytogenes,* as shown in Table 1.

**Figure 1:**
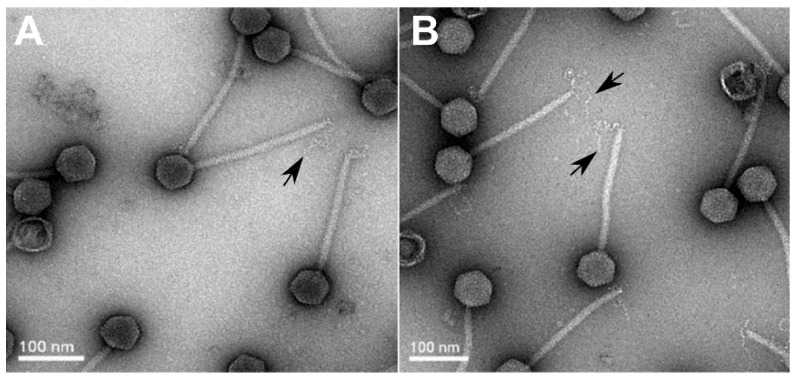
TEM-based designation of the *Salmonella* phages as Chi-like Siphoviruses. According to the isometric capsid (62 ± 5 nm) and non-contractile tail (220 ± 12 nm) morphologies, phages STm101 (**A**) and STm118 (**B**) are Siphoviruses. Black arrows designate the single, baseplate-attached tail fiber used by phage Chi and Chi-like phages for host attachment via interaction with the bacterial flagella [20,28,47].

TEM analysis revealed that the two phages belong to the *Siphoviridae* family, featuring isometric capsids (62 ± 5 nm) with long, non-contractile tails (220 ± 12 nm), as shown in Figure 1. Attached at the tip of each phage tail is the specialized receptor-binding protein (RBP)—a single, kinked tail fiber. The single tail fiber is a common feature among Chi-like phages and is used for the initial attachment to the flagella of host bacteria [20,21,22,28].

**Figure 2:**
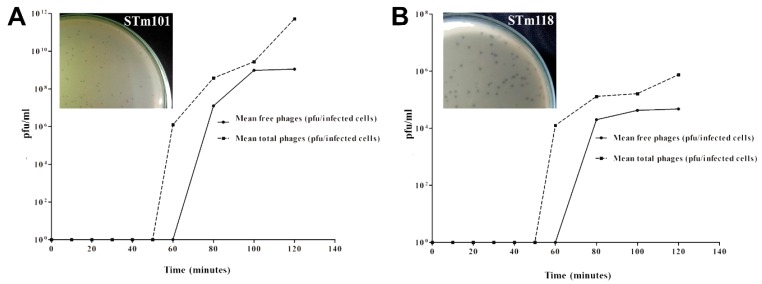
One-step growth curves of the *Salmonella* phages using a multiplicity of infection of 0.01. The mean free phages (solid line) and mean total phages liberated using chloroform (dotted line) are calculated as plaque forming units (pfu) for phages STm101 (**A**) and STm118 (**B**) infecting *S.* Tm MA8508. Shown as insets are pictures of the clear plaque morphologies for the two phages, STm101 and STm118 (**A** and **B**).

Data from one-step phage growth curves performed at MOI = 0.01 showed that phages STm101 and STm118 have similar growth patterns when using *S.* Tm MA8508 as a host, as shown in Figure 2. At ~60 min, the latent periods of both phages were extended more than the typical latent periods of 30 min observed for most *Salmonella* phages. STm118 also produced a reduced burst size (48 pfu/infected cell) compared to STm101 (112 pfu/infected cell) and other characterized *Salmonella* phages, which typically produce 100–230 pfu/infected cell [28,46]. However, these values depend heavily on the host and may change if tested using a different *Salmonella* host strain. Nevertheless, from the differences observed in their host ranges and plaque morphologies, we classified STm101 and STm118 as different phages. 

### 3.2. Genomic Analysis Identifies Thai Phage Isolates as Chi-Like Viruses

**Figure 3:**
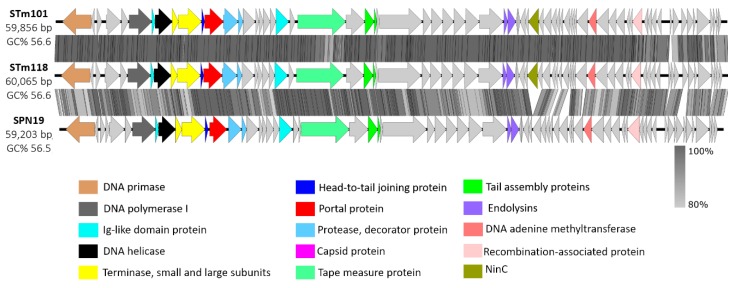
Alignment of *Salmonella* STm101, STm118, and the reference Chi-like SPN19 phage genomes based upon nucleotide sequence similarity, and aligned relative to their shared, terminal cos-sites at the left end. Predicted open reading frames (ORFs) are shown as arrows pointing in the direction of transcription and are color-coded according to their predicted functions.

The genomes of phages STm101 and STm118 are 59,856 and 60,065 bp, respectively, and share 98.8% nucleotide sequence similarity. Overall, the genomes of these phages are composed of 71 (STm101) and 72 (STm118) ORFs with a GC content of 56.6%, with no evident tRNAs, as shown in Supplementary Tables S4 and S5. These phages have approximately 93.6% to 96.2% nucleotide sequence identity to phage Chi (KM458633) and the Chi-like SPN19 (NC_019417), respectively, confirming both as members of the Chi-like virus genus. The phages all contain an integrase (*int*; gp59) with homology to integrases found in other Chi-like phages, e.g., gp14 in SPN19, suggesting a lysogenic lifestyle of these Chi-like phages. Moreover, during our studies we observed the formation of turbid plaques (a common trait of lysogenic phages) for STm101 and STm118 upon infection of *S.* Tm strain DB7155, providing evidence towards a lysogenic nature of these phages. As revealed in the phage Chi genome, both STm101 and STm118 feature the same cos-site (5′-GGTGCGCAGAGC-3’) at their genome termini, located 570 bps away from the first gene encoding a putative DNA primase [21]. As expected from their 98.8% sequence similarity, STm101 and STm118 present identical genomic organizations, with strong similarity to the Chi-like SPN19 phage (NC_019417.1), as shown in Figure 3. In brief, all three genomes can be aligned from the terminal cos-site follow by genes related to replication, i.e., those encoding putative DNA primase, polymerase, helicase, and the terminase subunits. However, as sequence variation between STm101 and STm118 was present across their entire genomes, determining a genetic link to their variable host ranges will require further investigation.

### 3.3. Phylogenomic Tree Inference of the Chi-Like Phage Cluster

Table 2 provides a comparison of our two phages to the 12 other members of the Chi-like cluster of *Salmonella*-infecting phages. The Chi-like cluster was identified among all *Salmonella* phages by hierarchically clustering all phage genomes according to their average amino acid identity, as shown in Supplementary Figure S1 and Supplementary Files 1 and 2. Of these, phages 35 and 37 encode the most genes, as shown in Table 2, despite being smaller than or the same size as the other phage genomes. These statistics are reflected in the average gene size for phage 35 and 37, which is 561 bp and 520 bp, respectively, compared to more than 700 bp for other genomes within the cluster. Furthermore, the genomes of phages 35 and 37 share the fewest genes with the rest of the genomes, with an average orthologous cluster size of 7.0 and 6.3, respectively, compared to 11.0–13.0 for the other phages. The gene content from genomes of the newly identified *Salmonella* phages STm101 and STm118 is relatively well conserved among genomes from the Chi-like phage cluster, as indicated by their large average cluster of orthologous group (COG) size. In addition, all genomes from the Chi-like phage cluster encode 28 orthologs, which can be used to infer phylogeny for the cluster, as shown in Supplementary File 3.

**Figure 4:**
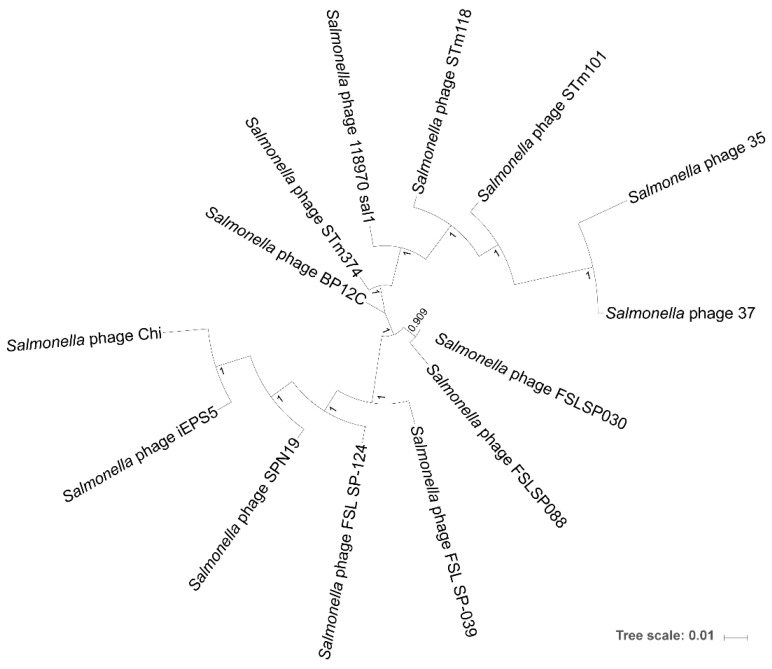
Phylogenomic tree representing the inferred evolutionary relationships of the *Salmonella* Chi-like phages. Local support values are calculated based on 1000 resamples using the Shimodaira–Hasegawa test on nearest-neighbor interchanges at each split. The tree was built from a concatenated codon-aware nucleotide alignment of 28 orthologs shared by the phages. The tree was built with FastTree (Version 2.1.10) using the generalized time reversible nucleotide substitution model.

Phage genomes are subjected to frequent horizontal gene transfer events that may obscure distant evolutionary relationships between them [50,51,52,53,54]. Additionally, they lack a universally conserved marker gene, which makes it difficult or impossible to determine phage phylogenies that extend deeply into evolutionary history, even with an abundance of phage genomes [52]. However, among small clusters of similar phage genomes, a core set of orthologs can allow us to infer the phylogenomic relationship among those phages. We identified 28 such orthologs for the Chi-like phages and inferred their phylogeny from the concatenated codon-aligned nucleotide sequences for these orthologs, as shown in Supplementary File 4. Internal nodes in the tree all have local support values (based on 1000 resamples of nearest-neighbor interchanges using the Shimodaira–Hasegawa test) of 100%, except for the node separating *Salmonella* phage FSL SP-030 and *Salmonella* phage FSL SP-088, which has a local support of 90.9%, as shown in Figure 4. Large local support values for every internal node suggested that this tree accurately represents the phylogeny of the Chi-like phages and that there is indeed a strong phylogenomic signal for the evolutionary history of this phage cluster. We did not root the tree due to the weak phylogenomic signal between the Chi-like cluster and other phage clusters. In agreement with the genome statistics in Table 2, the phylogenomic tree shows that phages 35 and 37 are the most distantly related to the rest of the cluster, meanwhile phages STm101 and STm118 form a distinct subclade with phages 118970 sal1, 35, and 37. The other major subclade is composed of phages FSL SP-039, FSL SP-124, SPN19, iEPS5, Chi, FSL SP-030, and FSL SP-088, while phage BP12C seems to be intermediately related to both major subclades, as shown in Figure 4.

### 3.4. Chi-Like Salmonella Phages Are Predominant among the Sampled Thai Poultry Farms

Genome sequence analysis identified the two phage isolates to be members of the Chi-like virus family. The quantification of Chi-like viruses across our 108 phage isolates was performed using PCR analysis with primers specific to the gene encoding virus capsid protein E [21,22]. Among 108 phages, 54 isolates, including STm101 and STm118, were positive as indicated by the presence of a 511 bp amplicon, as shown in Supplementary Figure S2. In addition, the percentage of Chi-like phage distribution between farms was not significantly different. Since all of these phages were isolated from chicken feces of four free-range farms in Northern Thailand and one commercial poultry farm in Central Thailand, we speculate that the Chi-like virus genus could be a predominant type among poultry farms in Thailand; however, wider sampling and further genetic analysis of *Salmonella* phage isolates across Thailand will be required to test this hypothesis.

### 3.5. Flagella and Lipopolysaccharide Are Required for Efficient Infection by the Two Thai Phages

Flagellotropic phages first infect motile bacterial hosts by attaching to the rotating flagella [20,26,28]. After flagella attachment, phages move down the flagella filament towards the body of the host where they bind irreversibly to secondary receptor(s) prior to DNA ejection into the host [55]. For example, phages ϕCb13 and ϕCbK use a pilus protein PilA as the secondary receptor for host attachment, with deletion of *pilA* (Δ*pilA*) providing complete resistance to the bacteria from phage infection [19]. Recently, it was also shown that phage 7-7-1, a flagellotropic *Myoviridae,* uses the LPS of its host *Agrobacterium* sp. as a secondary receptor for infection [27]. To the best of our knowledge, only the flagella has been identified for Chi-like phages as a receptor, with little known regarding potential secondary receptors. Thus, to identify other secondary receptors used by STm101 and STm118, we tested the ability of the phages to infect and lyse (assessed by plaque formation) nine strains of *S.* Tm deficient for various cell wall components proposed as phage receptors, as shown in Table 3. 

**Figure 5:**
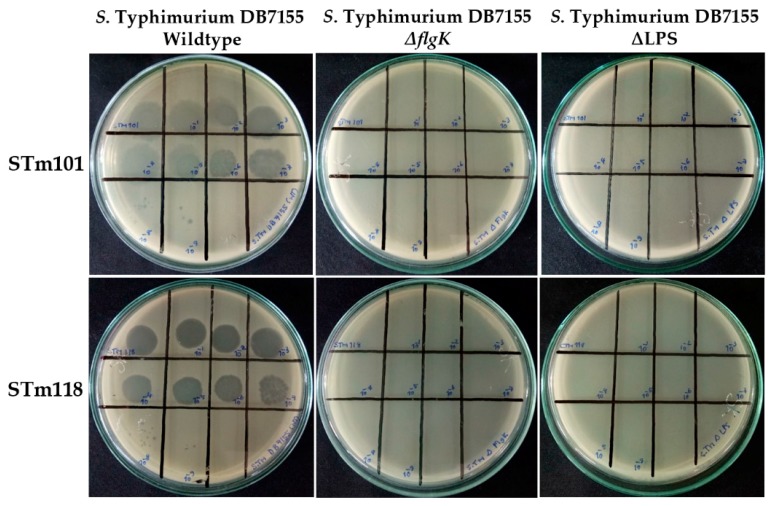
Spot tests of *Salmonella* phages STm101 and STm118 in serial dilution (10 μL of 10^11^–10^2^ pfu/mL) against lawns of *S*. Typhimurium wildtype, *ΔflgK,* and ΔLPS grown on trypticase soy agar (TSA). Clear zones of lysis and single plaque formation were only observed for both phages against *S*. Tm wildtype.

As expected for flagellotropic phages, plaque formation was inhibited for both phages against *S.* Tm *ΔflgK* (flagella hook protein deletion) compared to the wildtype strain, as shown in Figure 5. FlgK is a major component of the flagella, and therefore its deletion inhibits complete formation of this critical, primary receptor [28]. Plaque formation was not affected upon deletion of outer membrane porins (e.g., *ΔOmpC*) or molecular transporters (e.g., *ΔFadL*) compared to *S.* Tm wildtype and these were thus determined to not be receptors or necessary for host infection and lysis. Interestingly, both phages failed to produce plaques against *S.* Tm deficient in lipopolysaccharide (ΔLPS), as shown in Figure 5. This rough strain of *Salmonella* is completely devoid of carbohydrate substitutions to its KDO (2-keto-deoxy-d-octanoate) residues that form the core of the LPS [56]. While the LPS is a well-characterized receptor for many Gram-negative targeting phages [56,57,58,59,60], including the flagellotropic *Agrobacterium* phage 7-7-1 [27], this is the first study to suggest a role of the LPS for host infection by a member of the Chi-like phage genus.

## 4. Discussion

Rapid detection and remediation of *Salmonella* is essential for reducing the global burden associated with non-typhoidal *Salmonella* infections. Combined with increased emergence of multidrug resistant (MDR) *Salmonella*, innovative solutions are required to tackle the overuse of antibiotics in agriculture and food production [61,62], especially as these agents are often applied instead of proper hygiene measures. Phages have already been proven successful in reducing *Salmonella* contaminations in poultry [13,16], and for detection of food- and water-borne *Salmonella* [9,11,12].

*Salmonella enterica* is a highly diverse species of more than 2600 serovars [63]. In congruence to their highly diverse host, almost 250 *Salmonella* phages have so far been isolated and observed using electron microscopy [32]. In contrast, a smaller number of *Salmonella* phage genomes have been sequenced, meaning the study of *Salmonella* phage genomic diversity has remained rather limited. In this study, we isolated and characterized the biological and genomic properties of a diverse assortment of *Salmonella* phages isolated from poultry farms in Thailand. Host range analysis demonstrated that phages STm101 and STm118 could lyse approximately 50% of the 118 *Salmonella* tested strains. Unfortunately, the lysogenic lifestyle of this and other Chi-like phages is a major disadvantage for phage therapy, as virulence-associated genes could easily be spread among bacterial pathogens by phage integration [64]. However, genome-engineering strategies are rapidly advancing, and being applied to produce strictly lytic phages with adaptable host ranges or defined payloads [65] for clinical and diagnostic applications [66] including phage therapy in humans [67].

To investigate the genomic properties and diversity of the isolated *Salmonella* phages, the complete genomes of these phages were sequenced to identify them as members of the Chi-like virus genus. This also provides the first genomes of *Salmonella* Chi-like phages from Thailand, as shown in Table 2. PCR amplification of the Chi-like virus capsid protein E identified half of all phages isolated in this study as similar members of the Chi-like virus genus, suggesting that Chi-like phages could be a predominant phage family across these poultry farms. To explore the relationship between our isolated phages and previously reported Chi-like phages, we performed a phylogenomic analysis. Interestingly, phages STm101 and STm118 formed a monophyletic clade with phages isolated from Western countries, as shown in Figure 4; these included phage BP12C, isolated in USA (GenBank accession no. NC_031228) and phage 118970_sal1, isolated in Italy (GenBank accession no. NC_031930.1) [48], but not from closer isolated phages, such as phages SPN19 and iEPS5, isolated in Korea (GenBank accession no. NC_019417) [28]. However, further investigation and more phage isolates are required to investigate possible causes for this geographic distribution. Notably, the constructed phylogenomic tree was based on using the 28 core genes analysis and not the whole genome sequences. It may not reflect the relationships between the entire phages. Mavrich and Hatfull (2017) [68] reported that a set of core genes can be represented a phylogenomic relationship between phages only in the high-gene flux mode.

Initial recognition of the flagella by tail fibers of Chi-like phages is proposed as a mechanism to increase the likelihood of the phage identifying a motile and therefore metabolically active host that is suitable for progeny production [19,20]. Once the phage has navigated the length of the flagella to the cell body, it then attaches irreversibly to secondary receptors on the cell surface prior to triggered tail penetration and DNA ejection. Using *S.* Tm cells deficient in cell wall components, we identified the LPS as an essential component for efficient infection and lysis by phages STm101 and STm118. The LPS is composed of three distinct components—inner and outer cores and a hypervariable *O*-antigen—all of which are previously identified as receptors for phage adsorption. For example, *Salmonella* Podoviruses P22 and 9NA specifically recognize the repeating units of the *O*-antigen [10], meanwhile *Salmonella* phage S16 and *E. coli* phage T4 bind to the core LPS residues using two sets of tail fibers, which also triggers DNA ejection [56,69,70,71,72]. Identification of the specific LPS component recognized by the Chi-like phages, and how these are bound by the phage’s receptor-binding proteins, will be necessary to further our understanding of the infection process used by these unusual phages.

## Figures and Tables

**Table 1:** Host range infection of *Salmonella* phages STm101 and STm118.

*Salmonella* Serovar (s)	No. of Isolates	Spot Lysis (% Infectivity) of Phages	
		STm101	STm118
*S.* Enteritidis	32	3 (9.4%)	21 (65.6%)
*S.* Typhimurium	27	20 (74.1%)	17 (63%)
*S.* Virchow	15	7 (46.7%)	4 (26.7%)
*S.* Hadar	11	3 (27.3%)	2 (18.2%)
*S.* Choleraesuis	13	10 (76.9%)	6 (46.2%)
*S.* Agona	3	3 (100%)	3 (100%)
*S.* Saintpaul	1	1 (100%)	1 (100%)
*S.* Kentucky	1	0 (0%)	1 (100%)
*S.* Albany	3	0 (0%)	0 (0%)
*S.* I 4,[5],12:i:-	4	0 (0%)	0 (0%)
*S.* Altona	1	0 (0%)	0 (0%)
*S.* Corvallis	2	0 (0%)	0 (0%)
*S.* Give	3	0 (0%)	0 (0%)
*S.* Schwarzengrund	1	0 (0%)	0 (0%)
*S.* Singapore	1	0 (0%)	0 (0%)
**Total**	**118**	**47 (39.8%)**	**55 (46.6%)**
**Control strains**			
*Listeria monocytogenes*	13	0 (0%)	0 (0%)
*Vibrio* spp.	8	0 (0%)	0 (0%)
*Escherichia coli*	5	0 (0%)	0 (0%)
*Staphylococcus aureus*	5	0 (0%)	0 (0%)
**Total**	**31**	**0 (0%)**	**0 (0%)**

**Table 2:** Genome characteristics of *Salmonella* phages in the Chi-like phage cluster. CDSs: coding sequences; COG: cluster of orthologous group.

Phage	Accession no.	Size (bp)	GC (%)	CDSs	Avg CDS Size (bp)	tRNAs	Avg COG Size	Family	Bacterial Host	Source	Country	Reference
*Salmonella* phage STm101	KX765862	59,856	56.6	71	716	0	16.8	*Siphoviridae*	*S.* Typhimurium	Chicken feces	Thailand	This study
*Salmonella* phage STm118	KX765863	60,065	56.6	72	624	0	16.6	*Siphoviridae*	*S.* Typhimurium	Chicken feces	Thailand	This study
*Salmonella* phage STm374	KX765864	59,934	56.6	72	502	0	16.2	*Siphoviridae*	*S.* Typhimurium	Chicken feces	Thailand	-
*Salmonella* phage 118970_sal1	NC_031930.1	59,518	56.5	71	782	0	11.5	*Siphoviridae*	*S*. Enteritidis	Water buffalo feces	Italy	[48]
*Salmonella* phage 35	KR296689.1	55,391	56.8	91	561	0	7	*Podoviridae*	*S*. Gallinarum	Sewage	India	[49]
*Salmonella* phage 37	NC_029045.1	60,216	56.5	105	520	0	6.3	*Siphoviridae*	*S*. Gallinarum	Sewage	India	[49]
*Salmonella* phage BP12C	NC_031228.1	60,606	56.4	76	757	0	11.7	*Siphoviridae*	*S*. Hadar	Sewage	USA	-
*Salmonella* phage Chi	JX094499.1; KM458633.1	59,407	56.5	75	749	0	11.9	*Siphoviridae*	*Salmonella enterica, E. coli*	-	France	[21,22]
*Salmonella* phage FSL SP-039	KC139514	59,815	56.6	71	776	0	12.3	*Siphoviridae*	*S.* Cerro	Dairy farms	USA	[23]
*Salmonella* phage FSL SP-124	KC139515	59,245	56.5	71	765	0	12.2	*Siphoviridae*	*S*. Cerro	Dairy farms	USA	[23]
*Salmonella* phage FSL SP-030	NC_021779.1	59,746	56.6	71	776	0	12.3	*Siphoviridae*	*S*. Dublin	Dairy farms	USA	[23]
*Salmonella* phage FSL SP-088	NC_021780.1	59,454	56.4	70	779	0	12.3	*Siphoviridae*	*S.* Typhimurium	Dairy farms	USA	[23]
*Salmonella* phage iEPS5	NC_021783.1	59,254	56.3	73	765	0	12	*Siphoviridae*	*S*. Typhimurium	Sewage	South Korea	[28]
*Salmonella* phage SPN19	NC_019417.1	59,203	56.5	72	770	0	12.1	*Siphoviridae*	*S*. Typhimurium	Sewage	South Korea	-

**Table 3:** Results of spot lysis assays for STm101 and STm118 phage stocks were tested. The number of plaques formed are reported as being equivalent to wildtype (++), significantly reduced to wildtype (+), or no plaques formed (−).

*S.* Typhimurium	Other Designations and Features	Source	STm101	STm118
Wildtype	DB7155	[56]	++	++
Δ*ompA*	DB7155, *ΔompA*::Kan^r^; outer membrane protein A	[56]	++	++
Δ*ompC*	DB7155, *ΔompC*::Kan^r^; outer membrane protein C	[56]	++	++
Δ*ompX*	DB7155, *ΔompX*::Kan^r^; outer membrane protein X	[56]	++	++
Δ*btuB*	DB7155, *ΔbtuB*::Kan^r^; TonB-dependent vitamin B12 transporter	[56]	++	++
Δ*fadL*	DB7155, *ΔfadL*::Kan^r^; long-chain fatty acids transporter	[56]	++	++
Δ*tsx*	DB7155, *Δtsx*::Kan^r^; nucleoside-specific outer membrane channel	[56]	++	++
Δ*tonB*	DB7155, *ΔtonB*::Kan^r^; active transport regulator protein TonB	[56]	++	++
Δ*flgK*	SL1344_CH502, *ΔflgK*::Kan^r^; hook-filament junction protein	[28]	-	-
ΔLPS	DB7155 ΔLPS::Cm^r^; all LPS synthesis genes for regions distal to KDO residues deleted.	[56]	-	-

## Supplementary Materials

The following are available online at https://www.mdpi.com/1999-4915/11/6/520/s1, Figure S1: A hierarchically clustering all *Salmonella* phage genomes according to their average amino acid identity, Figure S2: 50% of all *Salmonella* phage isolates from Thai poultry farms are putative members of the Chi-like virus genus, Table S1: Complete list of *Salmonella* strains used for phage host range analysis title, Table S2: List of all identified *Salmonella* phages with the country of phage isolation, Table S3: Infectivity of other Chi-like phages against 4 serovars of *Salmonella enterica*, Table S4: List of *Salmonella* phage STm101 annotated ORFs and gene products, Table S5: List of *Salmonella* phage STm118 annotated ORFs and gene products, Supplementary file 1 (CSV file):Video Identified *Salmonella* phages with accession numbersS1: title, Supplementary File 2 (TSV file): Average amino acid identities of *Salmonella* phages, Supplementary file 3 (TSV file): A shared core set of 28 orthologs from the sequences of 14 Chi-like phages, Supplementary file 4 (FNA file): The concatenated codon-aligned nucleotide sequences of the 28 orthologs.

## Abbreviations

bp	base pair
gp	gene product
kbp	kilo-base pair
LPS	lipopolysaccharide
OM	outer membrane
ORFs	open reading frames
SM buffer	saline magnesium buffer
*S*. Tm	*S*. Typhimurium
PCR	polymerase chain reaction
COG	cluster of orthologous group
AAI	average amino acid identity
TEM	transmission electron microscopy
