# Supplementary material for: Characterization of Flagellotropic, Chi-Like Salmonella Phages Isolated from Thai Poultry Farms
Source: Viruses. 2019 Jun 5;11(6):520. doi: 10.3390/v11060520 (PMC6631126; doi:10.3390/v11060520)
Supplement: Supplementary file 1 [file viruses-11-00520-s001.zip › Phothaworn P. et al_Supplementary materials/Supplementary Information.docx]

**Supplementary Information**

**Supplementary File 1:** Identified *Salmonella* phages with accession numbers (CSV file 6.0 KB)**.**

**Supplementary File 2:** Average amino acid identities of *Salmonella* phages (TSV file 79 KB).

**Supplementary File 3:** A shared core set of 28 orthologs from the sequences of 14 Chi-like phages (TSV file 798 KB).

**Supplementary File 4:** The concatenated codon-aligned nucleotide sequences of the 28 orthologs (FNA file 223 KB).

**Supplementary Table S1:** Complete list of *Salmonella* strains used for phage host range analysis (XLSX file 13 KB).

**Supplementary Table S2:** List of all identified *Salmonella* phages with the country of phage isolation (XLSX file 16 KB).

**Supplementary Table S3:** Infectivity of other Chi-like phages against 4 serovars of *Salmonella* *enterica* (Word Document file 15 KB)*.*

**Supplementary Table S4:** List of *Salmonella* phage STm101 annotated ORFs and gene products (XLSX file 14 KB).

**Supplementary Table S5:** List of *Salmonella* phage STm118 annotated ORFs and gene products (XLSX file 15 KB).

**Supplementary Figure S1**

**

**

**Supplementary Figure S1**: A hierarchically clustering all *Salmonella* phage genomes according to their average amino acid identity.

**

Supplementary Figure S2**

**
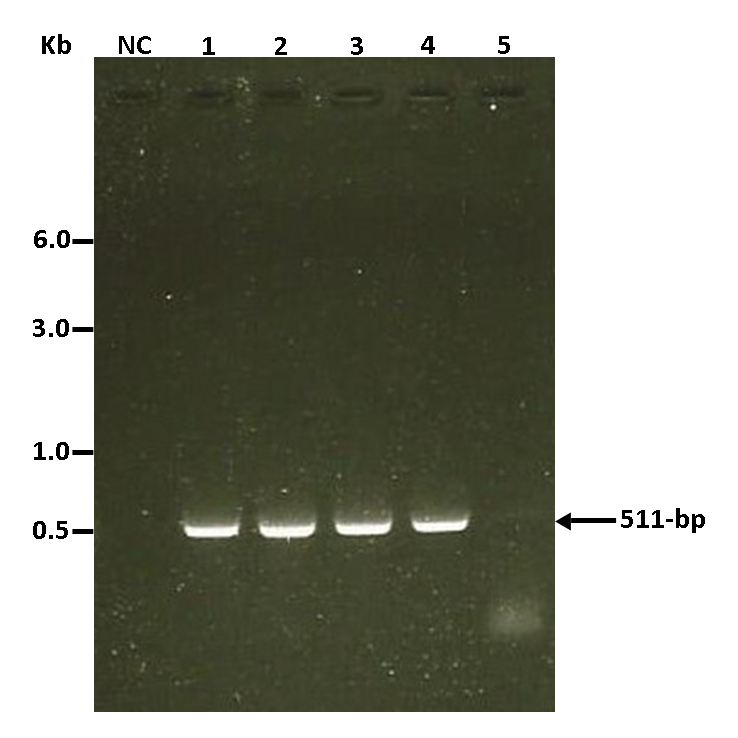
**

**Supplementary Figure S2:** 50% of all *Salmonella* phage isolates from Thai poultry farms are putative members of the Chi-like virus genus. The 511-bp PCR amplicon generated from phage DNA using primers specific for the Chi-like virus capsid protein E was visualized using 1% agarose gel electrophoresis. Positive amplification reactions are shown for STm101 (lane 1), STm118 (lane 2), STm374 (lane 3) and other Chi-like isolated phage (lane 4). Negative amplification reaction shown in lane 5 - suggesting an alternative phage type was isolated, or the primer-binding region of the capsid protein E gene sequence is different.
