# Supplementary material for: Characterization of Flagellotropic, Chi-Like Salmonella Phages Isolated from Thai Poultry Farms
Source: Viruses. 2019 Jun 5;11(6):520. doi: 10.3390/v11060520 (PMC6631126; doi:10.3390/v11060520)
Supplement: Supplementary file 1 [file viruses-11-00520-s001.zip › Phothaworn P. et al_Supplementary materials/Supplementary Table S3.docx]

**Supplementary Table S3**. Infectivity of other Chi-like phages against 4 serovars of *Salmonella* *enterica.*

| **No.** | **Phage** | **Spot lysis (%infectivity) of phages on *Salmonella* serovar (s)** | | | | |
| --- | --- | --- | --- | --- | --- | --- |
|  |  | **Typhimurium**  **(n = 12)** | **Hadar**  **(n = 6)** | **Virchow**  **(n = 6)** | **Enteritidis**  **(n = 20)** | **Overall % host range (n = 44)** |
| 1. | STm46C-1 | 4/12 (33.3%) | 0/6 (0%) | 1/6 (16.7%) | 1/20 (5.0%) | 6/44 (13.6%) |
| 2. | STm51C-1 | 8/12 (66.7%) | 0/6 (0%) | 4/6 (66.7%) | 0/20 (0%) | 12/44 (27.2%) |
| 3. | STm54C-1 | 4/12 (33.3%) | 0/6 (0%) | 3/6 (50.0%) | 2/20 (10.0%) | 9/44 (22.5%) |
| 4. | STm54C<1 | 5/12 (41.7%) | 0/6 (0%) | 4/6 (66.7%) | 0/20 (0%) | 9/44 (22.5%) |
| 5. | STm60C-2 | 8/12 (66.7%) | 0/6 (0%) | 4/6 (66.7%) | 0/20 (0%) | 12/44 (27.2%) |
| 6. | STm61C-1 | 8/12 (66.7%) | 0/6 (0%) | 3/6 (50.0%) | 0/20 (0%) | 11/44 (25.0%) |
| 7. | STm100C-1 | 9/12 (75.0%) | 0/6 (0%) | 2/6 (33.3%) | 6/20 (30.0%) | 17/44 (38.6%) |
| 8. | STm101C<1 | 6/12 (50.0%) | 0/6 (0%) | 3/6 (50.0%) | 0/20 (0%) | 9/44 (22.5%) |
| 9. | STm102C-1 | 7/12 (58.3%) | 0/6 (0%) | 2/6 (33.3%) | 3/20 (15.0%) | 12/44 (27.2%) |
| 10. | STm106C-1 | 7/12 (58.3%) | 0/6 (0%) | 0/6 (0%) | 11/20 (55.0%) | 18/44 (40.9%) |
| 11. | STm109C-1 | 9/12 (75.0%) | 0/6 (0%) | 3/6 (50.0%) | 2/20 (10.0%) | 14/44 (31.8%) |
| 12. | STm118C<1 | 9/12 (75.0%) | 0/6 (0%) | 0/6 (0%) | 0/20 (0%) | 9/44 (22.5%) |
| 13. | STm167C-1 | 4/12 (33.3%) | 0/6 (0%) | 1/6 (16.7%) | 0/20 (0%) | 5/44 (11.4%) |
| 14. | STm167C-2 | 5/12 (41.7%) | 0/6 (0%) | 2/6 (33.3%) | 0/20 (0%) | 7/44 (15.9) |
| 15. | STm167C<1 | 3/12 (25.0%) | 0/6 (0%) | 1/6 (16.7%) | 0/20 (0%) | 4/44 (9.1%) |
| 16. | STm363C-1 | 8/12 (66.7%) | 0/6 (0%) | 1/6 (16.7%) | 9/20 (45.0%) | 18/44 (40.9%) |
| 17. | STm364C-1 | 8/12 (66.7%) | 0/6 (0%) | 1/6 (16.7%) | 9/20 (45.0%) | 18/44 (40.9%) |
